# Supplementary material for: Selectivity for food in human ventral visual cortex
Source: Commun Biol. 2023 Feb 15;6:175. doi: 10.1038/s42003-023-04546-2 (PMC9932019; doi:10.1038/s42003-023-04546-2)
Supplement: Supplementary file 2 — Supplementary Material [file 42003_2023_4546_MOESM2_ESM.pdf]

Supplementary Materials

label	count
indoor	251
outdoor	693
ambiguous-location	54
plant	45
human-face	180
human-body	367
animal-face	142
animal-body	246
food	108
drink	25
food-related	130
faux-food	0
zoom	82
reach	80
large-scale-scene	833
object	551

Supplementary Table S1. Experiment 1. Count of the occurrence of each label across the 1,000 potentially shared images.

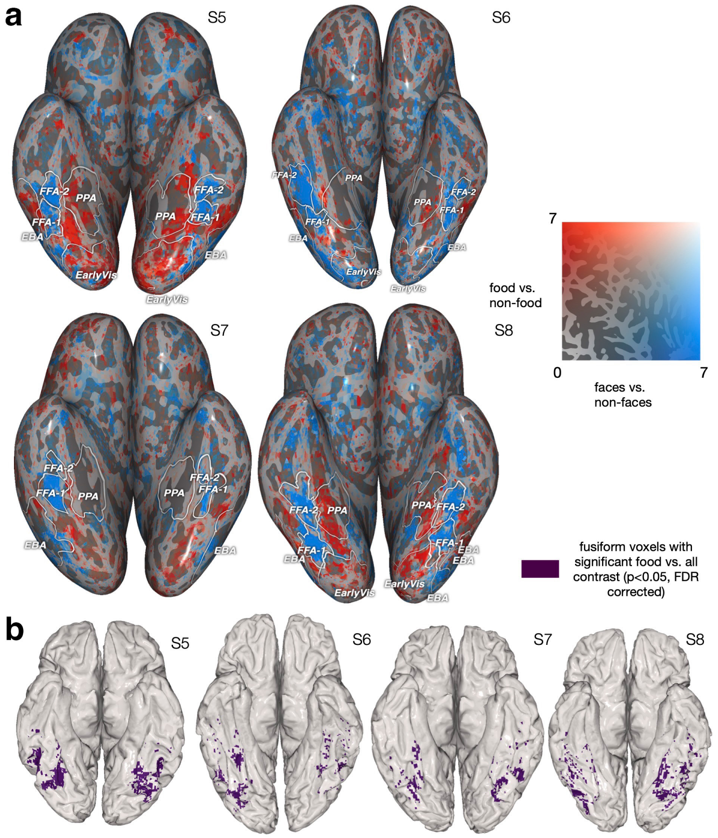

Supplementary Figure S1. ***Experiment 1.*** Voxels identified as selective for food for subjects S5-S8 shown on each subject's native surface with an inflated, bottom view (similar to Figure 2, but for different subjects) (a) Voxels' corresponding t-statistics from two 1-sided t-tests comparing food vs. non-food (red) and face vs. non-face (blue). Each t-test was performed on the weights from a trained OLS model, for example comparing the food label's learned weight against non-food labels' learned weights. The two sets of regions identified by each contrast are largely non-overlapping. This pattern is maintained when looking at food vs. non-(food and face) and face vs. non-(face and food) (Fig. S5). These results indicate that the two sets of regions have distinct activity for food and faces. (b) Spatial mask for food-selective regions used in subsequent analyses for S5-S8 (highlighting ventral visual responses). The mask is the overlap between the region that is identified from the t-test for food vs. non-food and relevant functionally localized regions using the HCP atlas [23] (see *Methods*).

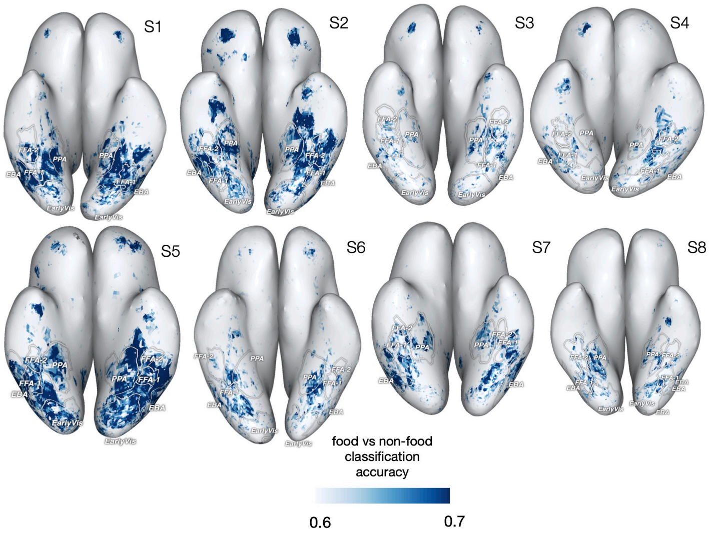

Supplementary Figure S2. ***Experiment 1.*** Classification accuracy for multivariate searchlight decoding food vs. non-food images for S1-S8, with darker voxels signifying higher accuracy. These regions encompass the two sets of regions corresponding to high values for the food vs. non-food and the face vs. non-face contrasts.

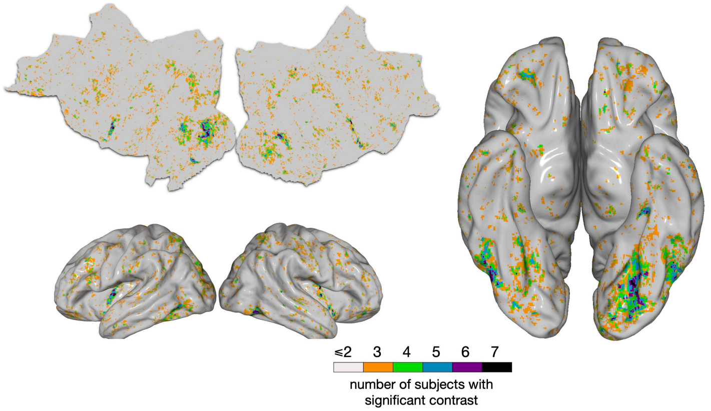

Supplementary Figure S3. ***Experiment 1.*** Semi-inflated bottom view of voxels, summed across S1-S8, that have significantly higher activity for the food than non-food categories, on the MNI surface, *considering only the non-reach images*. Significant voxels were identified similarly to Figure 1c.

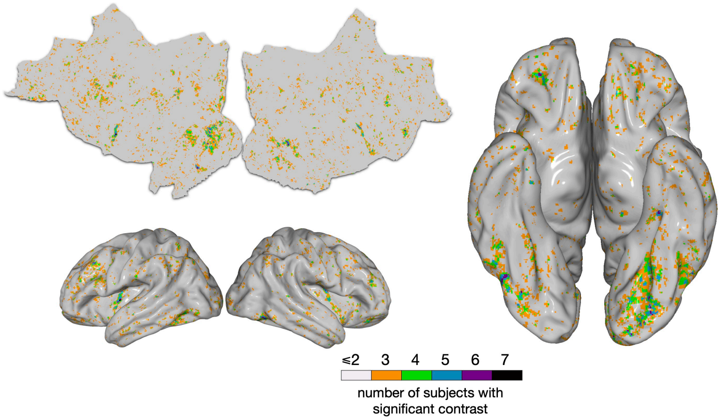

Supplementary Figure S4. ***Experiment 1.*** Semi-inflated bottom view of voxels, summed across S1-S8, that have significantly higher activity for the food than non-food categories, on the MNI surface, *considering only the non-zoom images*. Significant voxels were identified similarly to Figure 1c.

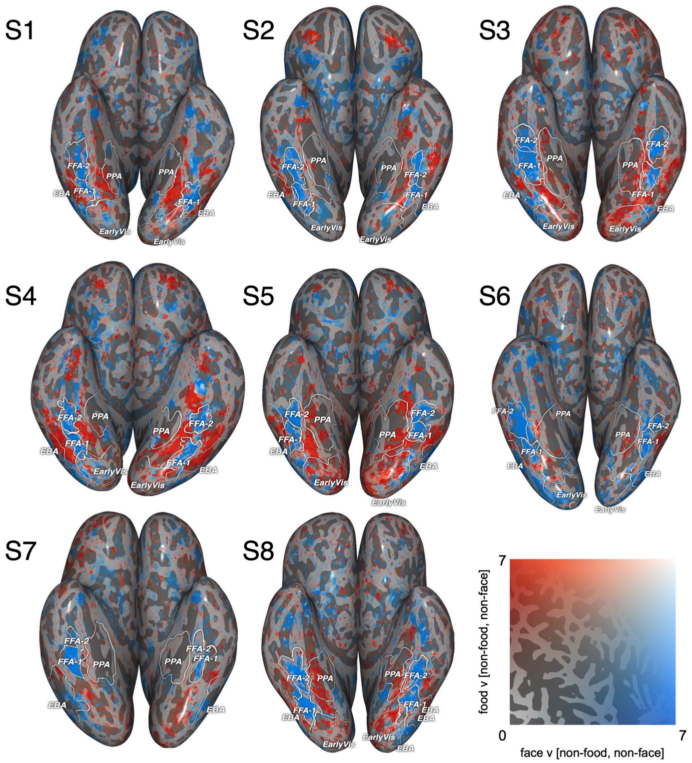

Supplementary Figure S5. ***Experiment 1.*** Voxels identified as selective for food from comparisons between food or faces vs. a baseline with *both* food *and* face removed for S1-S8. As described in Figure 2a, significant voxels were identified using two 1-sided t-tests. Despite a lower-N comparison arising from removing both faces and food from the baseline, there is still clear separability and little overlap between food-selective and face-selective regions.

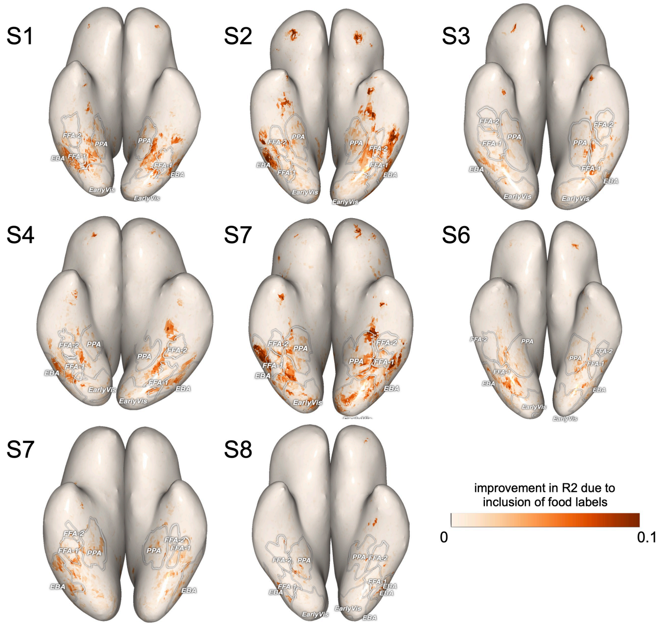

Supplementary Figure S6. ***Experiment 1.*** We compared predictive accuracy of an encoding model with all the COCO labels (including 13 food and 67 non-food labels) to an encoding model with only the 67 non-food COCO labels. The figure shows, for S1-S8, the improvement in validation set R^2 values when including the food labels (R^2 for the full model - R^2 for the model with food removed).

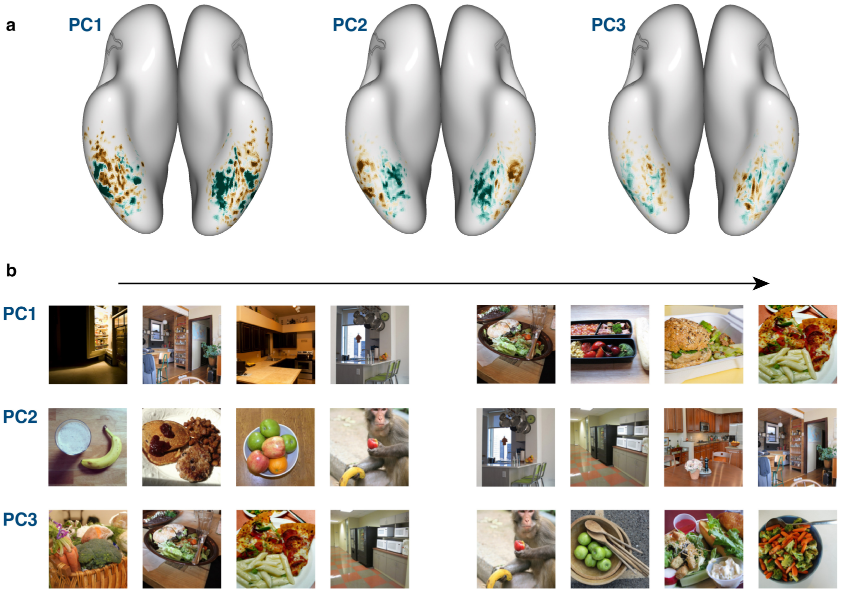

Supplementary Figure S7. Experiment 1. PCA of responses from food-selective regions excluding images containing human faces and human bodies. (a) Average principal component score across subjects for PC1, PC2, and PC3, shown on the MNI surface. Blue-green indicates high, brown indicates low PC scores. In (b) we plot the top and bottom images for PC1, PC2, and PC3 along a linear axis (lowest to highest from left to right). We include the 4 lowest and 4 highest images for ease of visualization. The patterns across the brain that emerge here are remarkably well aligned with the patterns seen for the full PCA (Figure 4a). Qualitatively, PC1 and PC2 again seem to distinguish large-scale images of food-related places from close-by images of food, as well as capturing the prominence of food in an image, separating images with focus on food in the foreground from those with food in the background. PC3, however, appears less interpretable and does not support any useful inferences.

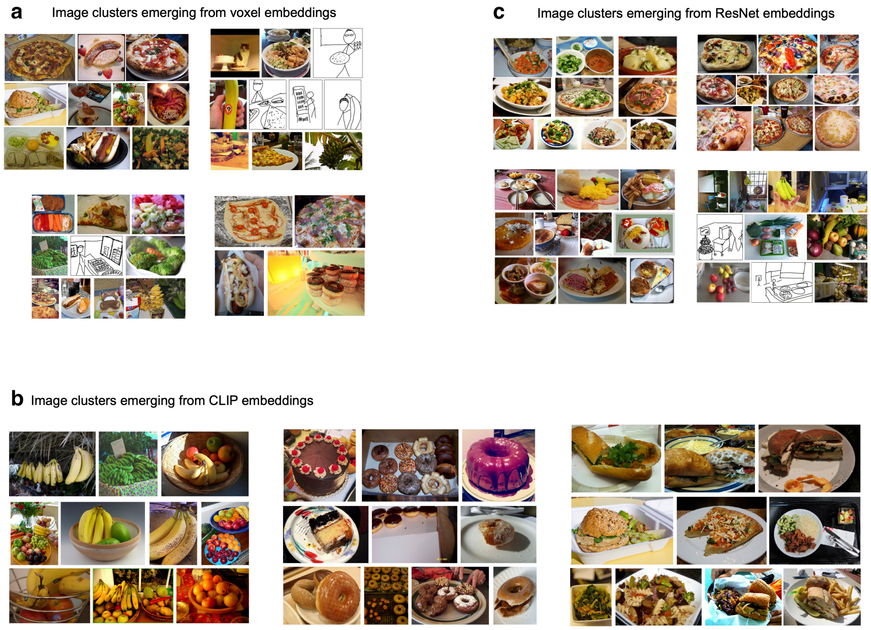

Supplementary Figure S8. ***Experiment 1.*** (a) Image clusters based on voxel-response embeddings for S1. (b) Image clusters based on CLIP embeddings. The clusters appear to capture semantic properties such as fruit or baked goods. (c) Image clusters based on ResNet-18 embeddings. The clusters appear to capture visual properties such as color (e.g., green and orange), global shape (e.g., round), or image complexity. The two neural-network-derived clustering patterns show little to no correlation with the brain-derived clusters (the Voxel-CLIP correlation being 0.030; the Voxel-Resnet-18 correlation being 0.026 – both being lower than the CLIP-Resnet-18 correlation of 0.256). This suggests that food-selective regions are organized on the basis of features absent from deep layers of typical high-performing neural networks. Due to licensing concerns, COCO images showing people that were used in our study have been replaced with stick figures representing the structure of the original stimulus images. Replacement images (badly) drawn by MJT.

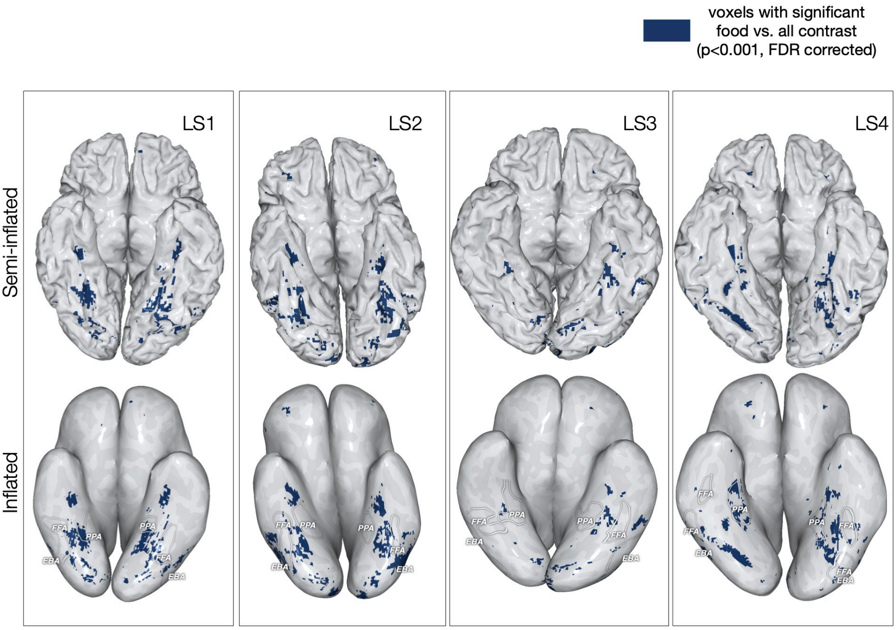

Supplementary Figure S9. ***Experiment 2.*** Voxels identified as selective for food for subjects LS1-LS4 shown on each subject's native surface with a semi-inflated, bottom view [Top] and an inflated view [Bottom]. Voxels were identified as selective by testing for the significance of the contrast (p <0.001, FDR corrected).

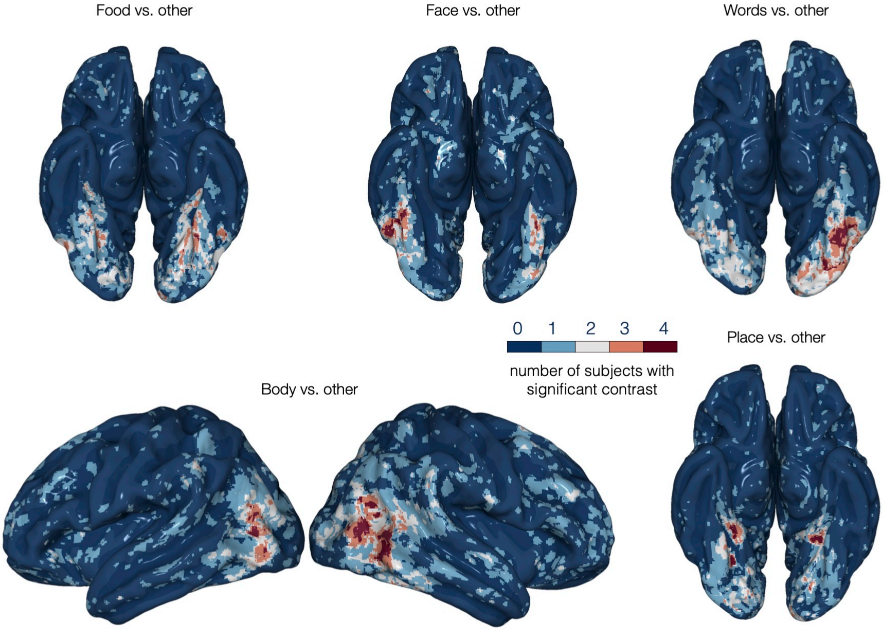

Supplementary Figure S10. ***Experiment 2.*** Semi-inflated bottom view of voxels, summed across LS1-LS4, that have significantly higher activity for the different contrasts in Experiment 2, on the MNI surface. Significant voxels were identified similarly to Fig. 5.

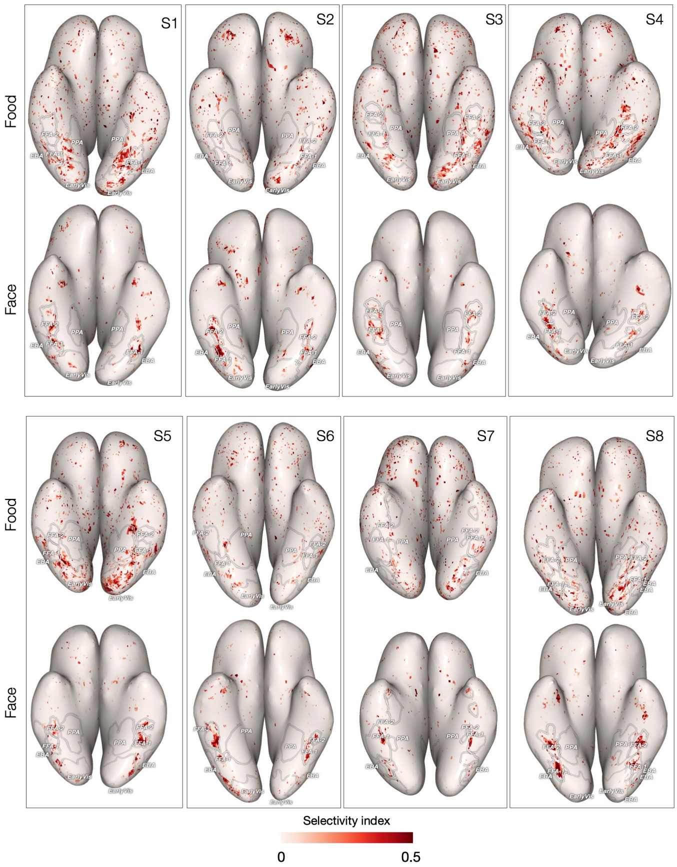

Supplementary Figure S11. ***Experiment 1.*** **Voxel-wise selectivity for food and faces viewed in natural scene images.** Selectivity was defined as: $\frac{\text{preferred - non-preferred}}{|\text{preferred }|+|\text{non-preferred}|}$ where the non-preferred baseline activity is the *maximum* activity related to any other category. This is a *conservative* and less biased measure of selectivity in that we consider the food or face response of each voxel relative to the highest response across all other possible categories for that voxel (rather to a single fixed baseline category). This approach allows for voxel by voxel variation in selectivity rather than assuming that an entire region's voxels respond in an uniform manner. To measure selectivity for Category c, we compute $\frac{\beta_c - \max_{i,i\neq c}\beta_i}{|\beta_c|+|\max_{i,i\neq c}\beta_i|}$, where β_i is the weight of the OLS encoding model corresponding to Category i. Selective voxels for each category are plotted on inflated views of individual subjects' brains. To better visualize the largest number of category selective voxels, positive selectivity values greater than 0.5 and negative selectivity values are not plotted (because negative selectivity denotes non-preferred category-selective responses that obscure the preferred category-selective regions; e.g., when measuring food selectivity for voxels in the FFA, the highest response for a non-food category was typically for faces and this response was typically higher than for food for that voxel, thereby producing a negative food selectivity index).

a

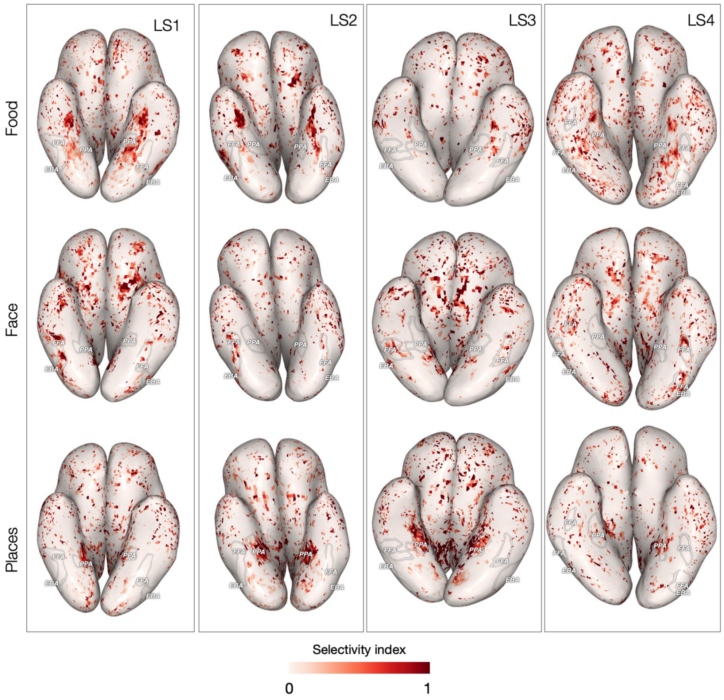

b

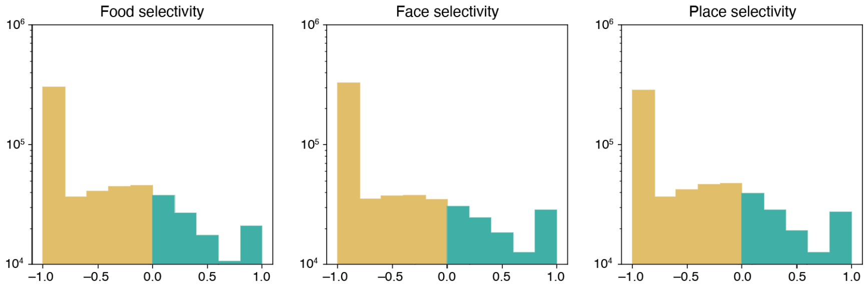

Supplementary Figure S12. ***Experiment 2.*** **Voxel-wise selectivity for food, faces and places viewed in the localizer images.** As in Figure S11, selectivity was defined as: $\frac{\text{preferred - non-preferred}}{|\text{preferred }|+|\text{non-preferred}|}$ where the non-preferred baseline activity is the *maximum* activity related to any other category. To measure selectivity for Category c, we compute $\frac{\beta_c - \max_{i,i\neq c}\beta_i}{|\beta_c|+|\max_{i,i\neq c}\beta_i|}$, where β_i is the weight of the OLS encoding model corresponding to Category i. (a) Selective voxels for each preferred category are plotted on inflated views of individual subjects' brains. Negative selectivity values are not included on the brain maps because they obscure the preferred category-selective regions. (b) The distribution of selectivity values reveals roughly equal preference strength for food, faces, and places. Voxels showing selectivity for the category being measured are plotted in aqua, while voxels showing negative selectivity for that category are plotted in gold – typically representing voxels in other category-selective regions.
