# Supplementary material for: Selectivity for food in human ventral visual cortex
Source: Commun Biol. 2023 Feb 15;6:175. doi: 10.1038/s42003-023-04546-2 (PMC9932019; doi:10.1038/s42003-023-04546-2)
Supplement: Supplementary file 3 — Reporting Summary [file 42003_2023_4546_MOESM3_ESM.pdf]

nature portfolio

Corresponding author(s): Michael J Tarr
Leila Wehbe

Last updated by author(s): Jan 5, 2023

Reporting Summary

Nature Portfolio wishes to improve the reproducibility of the work that we publish. This form provides structure for consistency and transparency in reporting. For further information on Nature Portfolio policies, see our Editorial Policies and the Editorial Policy Checklist.

Statistics

For all statistical analyses, confirm that the following items are present in the figure legend, table legend, main text, or Methods section.

n/a	Confirmed	
☐	☒	The exact sample size (*n*) for each experimental group/condition, given as a discrete number and unit of measurement
☐	☒	A statement on whether measurements were taken from distinct samples or whether the same sample was measured repeatedly
☐	☒	The statistical test(s) used AND whether they are one- or two-sided<br>*Only common tests should be described solely by name; describe more complex techniques in the Methods section.*
☐	☒	A description of all covariates tested
☐	☒	A description of any assumptions or corrections, such as tests of normality and adjustment for multiple comparisons
☐	☒	A full description of the statistical parameters including central tendency (e.g. means) or other basic estimates (e.g. regression coefficient) AND variation (e.g. standard deviation) or associated estimates of uncertainty (e.g. confidence intervals)
☐	☒	For null hypothesis testing, the test statistic (e.g. *F*, *t*, *r*) with confidence intervals, effect sizes, degrees of freedom and *P* value noted<br>*Give P values as exact values whenever suitable.*
☒	☐	For Bayesian analysis, information on the choice of priors and Markov chain Monte Carlo settings
☒	☐	For hierarchical and complex designs, identification of the appropriate level for tests and full reporting of outcomes
☒	☐	Estimates of effect sizes (e.g. Cohen's *d*, Pearson's *r*), indicating how they were calculated

Our web collection on statistics for biologists contains articles on many of the points above.

Software and code

Policy information about availability of computer code

Data collection	Python, Matlab, Psychtoolbox
Data analysis	Python, Freesurfer, Pycortex

For manuscripts utilizing custom algorithms or software that are central to the research but not yet described in published literature, software must be made available to editors and reviewers. We strongly encourage code deposition in a community repository (e.g. GitHub). See the Nature Portfolio guidelines for submitting code & software for further information.

Data

Policy information about availability of data

All manuscripts must include a data availability statement. This statement should provide the following information, where applicable:

- Accession codes, unique identifiers, or web links for publicly available datasets
- A description of any restrictions on data availability
- For clinical datasets or third party data, please ensure that the statement adheres to our policy

The NSD data is available as detailed by Allen et al.Nat. Neurosci. 25(1) (2022). The localizer data is found in the link below and will be made available upon publication:
https://drive.google.com/drive/folders/1gSrRNibSIJ9RS9tydsOGssy4fCvs aEw

Human research participants

Policy information about studies involving human research participants and Sex and Gender in Research.

Reporting on sex and gender	Data on the sex of the participants for NSD is as reported in Allen et al.Nat. Neurosci. 25(1) (2022). For our localizer, sex information was collected for demographic purposes only. Sex is not a relevant factor within our study, so no further use of this information was made other than reporting it for purposes of characterizing our participants.
Population characteristics	Data on the age range of the participants for NSD is as reported in Allen et al.Nat. Neurosci. 25(1) (2022). No age data was collected for our localizer. No other data regarding participant demographics was collected in our localizer.
Recruitment	Participants were recruited from the University of Minnesota community for the NSD study (see Allen et al.Nat. Neurosci. 25(1) (2022)) and from the Carnegie Mellon community for our localizer.
Ethics oversight	University of Minnesota IRB (NSD) and Carnegie Mellon IRB (localizer).

Note that full information on the approval of the study protocol must also be provided in the manuscript.

Field-specific reporting

Please select the one below that is the best fit for your research. If you are not sure, read the appropriate sections before making your selection.

☒ Life sciences ☐ Behavioural & social sciences ☐ Ecological, evolutionary & environmental sciences

For a reference copy of the document with all sections, see nature.com/documents/nr-reporting-summary-flat.pdf

Life sciences study design

All studies must disclose on these points even when the disclosure is negative.

Sample size	No sample size calculation was performed in either study.
Data exclusions	No data was excluded.
Replication	The results of our NSD study have been replicated in two other studies from other research groups. Experiment 2 has not been replicated.
Randomization	All participants viewed a random subsample of images (see Allen et al.Nat. Neurosci. 25(1) (2022)) or all images (localizer). There was no allocation of participants to different conditions.
Blinding	Since there was no group allocation for participants, no blinding was necessary or appropriate.

Reporting for specific materials, systems and methods

We require information from authors about some types of materials, experimental systems and methods used in many studies. Here, indicate whether each material, system or method listed is relevant to your study. If you are not sure if a list item applies to your research, read the appropriate section before selecting a response.

Materials & experimental systems

n/a	Involved in the study	
☒	☐	Antibodies
☒	☐	Eukaryotic cell lines
☒	☐	Palaeontology and archaeology
☒	☐	Animals and other organisms
☒	☐	Clinical data
☒	☐	Dual use research of concern

Methods

n/a	Involved in the study	
☒	☐	ChIP-seq
☒	☐	Flow cytometry
☐	☒	MRI-based neuroimaging

Magnetic resonance imaging

Experimental design

Design type	functional MRI. See Allen et al.Nat. Neurosci. 25(1) (2022) for design details on the NSD studyo. Our localizer used a standard blocked design.
Design specifications	See Allen et al.Nat. Neurosci. 25(1) (2022) for details on the design used for NSD. For our localizer, we data was collected in runs of length 4 minutes (360 images per run). Subject LS1 underwent 4 runs of the localizers, while subjects LS2-LS4 underwent 9 runs.
Behavioral performance measures	NSD: Image memory task (has the image been shown before?).<br>Localizer: 1-back repeated item detection.

Acquisition

Imaging type(s)	functional, structural
Field strength	NSD: 7T; Experiment 2: 3T
Sequence & imaging parameters	NSD: The functional MRI data were acquired at 7T using whole-brain gradient-echo EPI at 1.8-mm resolution and 1.6s repetition time.<br>Localizer: Functional images were collected using a T2*-weighted gradient recalled echoplanar imaging multi-band pulse sequence (cmrr mbep2d bold) from the University of Minnesota Center for Magnetic Resonance Research (CMRR) [64, 65]. Parameters: 68 oblique axial slices co-planar with the AC/PC; in-plane resolution=2×2mm; 106×106 matrix size; 2mm slice thickness, no gap; interleaved acquisition; field of view=212mm; phase partial Fourier scheme of 6/8; TR=1500 ms; TE=30ms; flip angle=79 degrees; bandwidth=1814 Hz/Px; echo spacing=0.68ms; excite pulse duration=8200 microseconds; multi-band factor=4; phase encoding direction=A to P; fat saturation on; advanced shim mode on.
Area of acquisition	Whole brain.

Diffusion MRI ☐ Used ☒ Not used

Preprocessing

Preprocessing software	For details on NSD preprocessing see Allen et al.Nat. Neurosci. 25(1) (2022). For the localizer each subject's native surface was reconstructed using Freesurfer. Functional scans were motion corrected using SPM12.
Normalization	For details on NSD see Allen et al.Nat. Neurosci. 25(1) (2022). For the localizer, through Pycortex, alignment of the functional data to the structural data was obtained (using bbregister from Freesurfer). Pycortex was used to mask the cortical data (by relying on maps estimated by Freesurfer). Pycortex was also used for transformation to MNI space. It relies on the Flirt tool from FSL.
Normalization template	MNI
Noise and artifact removal	For details on NSD see Allen et al.Nat. Neurosci. 25(1) (2022). For the localizer our code pipeline (provided in the folder referred to in the Code Availability Statement section) includes detrending and lightly smoothed with a Gaussian kernel of standard deviation 1mm, using standard functions part of the scipy package.
Volume censoring	N/A

Statistical modeling & inference

Model type and settings	For NSD we utilized voxel-wise ordinary least squares (OLS) encoding models to predict each individual voxel response to a given stimulus. Identifying voxels more responsive to category A over other category was done using a 1-sided t-test between the respective learned model coefficients for category A vs. the coefficients for the other categories, as is done in a typical generalized linear model (GLM) analysis. Decoding models were implemented as in the Methods and Materials section of our paper.<br>For the localizer we computed a contrast between condition A and other conditions after estimating voxel-wise ordinary least squares (OLS) encoding models.
Effect(s) tested	For NSD we identified voxels that are more responsive to food than other labels, as well as for face versus other labels. We obtained a p-value from the t-value, then corrected for multiple comparisons.<br>For the localizer we computed a t-value for each of the "food vs. other", "face vs. other", "body vs. other", "place vs. other" and "word vs. other" contrasts. We obtained a p-value from the t-value, then corrected for multiple comparisons.

Specify type of analysis: ☐ Whole brain ☒ ROI-based ☐ Both

Anatomical location(s)	ventral visual cortex
Statistic type for inference (See Eklund et al. 2016)	voxel-wise encoding models, decoding, one-side t-tests
Correction	For both NSD and the localizer we used the Benjamini-Hochberg False Discovery Rate procedure (FDR) and alpha = 0.05 to identify significant voxels for each contrast of each subject at each voxel.

Models & analysis

n/a	Involved in the study
☒	☐ Functional and/or effective connectivity
☒	☐ Graph analysis
☐	☒ Multivariate modeling or predictive analysis

Multivariate modeling and predictive analysis	For NSD we used encoding models, decoding models, Principal Component Analysis, and k-means clustering as detailed in our Methods and Materials.<br>For the localizer we used encoding models as well as calculating a voxel-wise selectivity index. In all cases the dependent variable is the voxel-wise BOLD response. The independent variables were the visual and semantic content of the images used as stimuli, including specific categories contained in those images (i.e., food, faces, people, places, and scenes).
